# Impact of Add‐On Hepatobiliary Abbreviated Magnetic Resonance Imaging on Ultrasound Hepatoma Surveillance for Liver Cirrhosis‐ a Randomized Study

**DOI:** 10.1002/kjm2.70104

**Published:** 2025-09-01

**Authors:** Jing‐Houng Wang, Hsin‐You Ou, Yi‐Hao Yen, Chao‐Hung Hung, Sheng‐Nan Lu

**Affiliations:** ^1^ Department of Internal Medicine Kaohsiung Chang Gung Memorial Hospital Kaohsiung City Taiwan; ^2^ Department of Radiology Kaohsiung Chang Gung Memorial Hospital Kaohsiung City Taiwan

**Keywords:** abbreviated magnetic resonance imaging (AMRI), gadoxetic acid (GA), hepatocellular carcinoma (HCC) surveillance, liver cirrhosis, ultrasound (US)

## Abstract

Hepatocellular carcinoma (HCC) surveillance with semi‐annual ultrasound (US) is recommended for high‐risk patients. This study investigates the impact of hepatobiliary abbreviated magnetic resonance imaging (AMRI) performed annually on the recommended US surveillance. Patients with compensated liver cirrhosis at regular HCC surveillance using US and alpha‐fetoprotein, with adequate renal function and without HCC diagnosis, were enrolled. Patients were randomized into add‐on hepatobiliary AMRI and continuous US surveillance groups. For patients in the AMRI group, gadoxetic acid‐enhanced AMRI was performed at enrollment and annually. Liver nodule detection, HCC diagnostic tests, and HCC development were compared between the two groups. One hundred and four patients were initially enrolled, with 15 patients excluded for loss of regular follow‐up, giving a total of 89 patients (AMRI: 45 and US: 44) that were analyzed in a median follow‐up of 33.6 months. There were no significant differences in baseline characteristics nor statistical differences in hepatic nodule detections (AMRI:10 vs. US:18, *p* = 0.074) and HCC developments (1 vs. 6, *p* = 0.058) between the groups. While one HCC with a size of 1.2 cm (BCLC stage:0) was diagnosed in the AMRI group, six HCCs with a mean size of 2.4 cm (BCLC stage 0:2, A:3, B:1) were found in the US group. Compared with the AMRI group, there were more patients in the US group (18 vs. 9, *p* = 0.032) underwent dynamic imaging and/or biopsy. Curative treatments were performed for all patients with HCC. For compensated cirrhosis patients in the recommended US surveillance, hepatobiliary AMRI annually might reduce the frequency of HCC diagnostic tests.

**Abbreviations**
AFP: alpha‐fetoprotein
AMRI: abbreviated magnetic resonance imaging
DWI: diffusion‐weighted image
GA: gadoxetic acid
HBP: hepatobiliary phase
HCC: hepatocellular carcinoma
IQR: interquartile range
SD: standard deviation
US: ultrasound

## 1 Introduction

Hepatocellular carcinoma (HCC) is the major cancer mortality in many countries and is predicted to increase by 55% between 2020 and 2040 [1]. Much progress with regard to HCC surveillance and treatment has led to improvement in patient survival, where HCC surveillance in tertiary hospitals or community settings with appropriate treatment has been demonstrated to improve survival for high‐risk patients of HCC [2, 3]. For patients with cirrhosis, HCC surveillance is associated with early‐stage HCC detection, curative treatment receipt, and survival [4]. Professional societies have recommended HCC surveillance for high‐risk populations using ultrasonography (US) with/without alpha‐fetoprotein (AFP) at a 3‐ to 6‐monthly intervals [4, 5]. Although US is the recommended modality, it is an operator‐dependent procedure and limited by low sensitivity in early‐stage HCC detection, which was 47% in one meta‐analysis [6]. In addition, US quality has been determined as inadequate to exclude HCC in 20.3% of patients and changes dynamically, especially for obese patients and those with alcoholic or nonalcoholic fatty liver disease‐related cirrhosis [7, 8]. New imaging modalities or techniques with high sensitivity for early‐stage HCC detection might have potential in improving the effectiveness of HCC surveillance and achieving the concept of precision surveillance [9].

Magnetic resonance image (MRI) is an emerging imaging technique with high sensitivity and specificity for HCC surveillance; however, it is limited by high cost, lengthy exam time, and radiological capacity [9]. To save exam time and reduce cost, abbreviated MRI (AMRI) with a few essential sequences has been proposed to overcome these limitations. Although most studies have been retrospective and not truly representative of screening populations, the pooled per‐patient sensitivity and specificity of AMRI was 86% and 94% in one meta‐analysis [10], and semiannual surveillance with MRI was cost‐effective in cirrhotic patients with yearly HCC incidence ⩾ 1.81% or 3% [11, 12]. AMRI might reinforce cost‐effectiveness by reducing acquisition and interpretation time [12, 13]. Semiannual AMRI for high‐risk HCC, AMRI every 12 months, and semiannual US have been suggested for patients with intermediate HCC risk [14]. Before applying such suggestions in clinical practice, however, it is important to investigate the benefits and risks of AMRI on HCC surveillance using US for high‐risk populations.

The purpose of this randomized study was to assess the impact of add‐on hepatobiliary AMRI performed annually on HCC surveillance with recommended US at an interval of three to 6 months for high‐risk patients.

## 2 Materials and Methods

### 2.1 Patients

This study protocol was approved by the Institutional Review Board of Chang Gung Memorial Hospital. All patients signed informed consents before enrollment; specifically, patients with compensated liver cirrhosis in liver function reserve Child‐Pugh A classification and in HCC surveillance protocol using US were enrolled. Liver cirrhosis was diagnosed as liver stiffness ⩾ 15 kPa by transient elastography, US, or histology cirrhosis with liver stiffness ⩾ 10 kPa, or US cirrhosis with varices. Patients with histories of decompensation were excluded, including those with conditions such as variceal bleeding, ascites, and hepatic encephalopathy, along with those having histories of malignancy, serum creatinine level > 1.5 mg/dL, or history of MRI contrast allergy or claustrophobia. With computer‐assisted methods, a randomization sheet was generated with patients randomized according to the randomization number at the time of enrollment. The enrolled patients were randomized 1:1 into separate AMRI group and US groups. Patients in the AMRI group received add‐on hepatobiliary AMRI at enrollment and annually, in addition to US surveillance every 3–6 months. Patients in the US group underwent HCC surveillance with US every 3–6 months. For all enrolled patients, AFP levels were also determined every 3–6 months. Further investigations of abnormal US findings were based on the overall judgment according to the clinical practice of the physician. The diagnosis of HCC is based on typical dynamic imaging findings (enhancement on arterial phase and washout on portal/delayed phase) or histology/cytology according to professional guidelines of HCC management [4, 5]. HCC treatment was based on multi‐disciplinary discussion. All patients were followed up until HCC diagnosis, death, or the end of the study.

### 2.2 Add‐On Hepatobiliary AMRI

AMRI was performed with a 1.5‐T whole‐body MRI system (Discovery MR450, GE. Healthcare, Milwaukee, WI) and an eight‐channel controlled‐array software coil. After overnight fasting, 10 mL gadoxetic acid (GA) (Bayer Pharm AG, Germany) was administered intravenously (0.25 mmol/mL) 10–20 min before AMRI. Hepatobiliary AMRI sequences consisted of diffusion‐weighted imaging (DWI), T2‐weighted and delayed hepatobiliary phase imaging. The exam time was less than 10 min, with suspicion of HCC based on hepatobiliary AMRI as follows: (1) On DWI: when a lesion showed restricted diffusion; (2) On T1w‐hepatobiliary phase (HBP): when a lesion showed hypointensity; and (3) On full AMRI (DWI + T1w‐HBP combined), when a nodule showed restricted diffusion and hypointensity on T1w‐HBP. Hepatobiliary AMRI images were interpreted and reported in a structured form by one experienced liver radiologist (Ou HY).

### 2.3 Statistical Analysis

Continuous variables were expressed as mean with standard deviation (SD) and median with interquartile range (IQR). Categorical data was expressed as absolute and relative frequencies. Student *t‐test* was used to compare continuous data, while χ^2^ or Fishe's exact tests were used to compare categorical variables. The cumulative incidence of hepatic nodule detection and HCC was analyzed with the Kaplan–Meier method and log‐rank test.

## 3 Results

### 3.1 Patients

**FIGURE 1:**
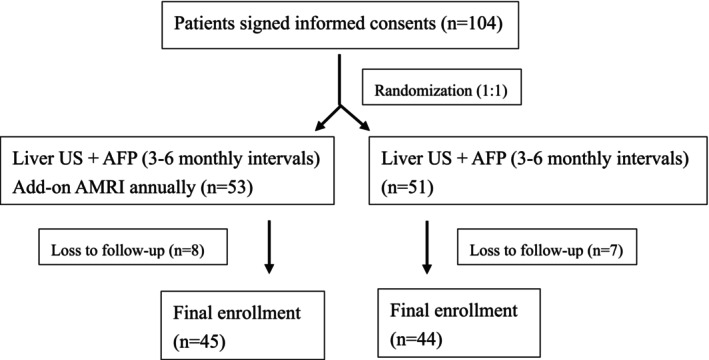
The flowchart of patient enrollments. The enrolled patients were randomized into ultrasound (US) and add‐on hepatobiliary abbreviated magnetic resonance imaging (AMRI) groups.

One hundred and four patients signed informed consents and were randomized into AMRI and US groups. Fifteen patients including eight in the AMRI group and seven in the US group were excluded due to loss of follow‐up. Finally, there were 45 and 44 patients enrolled in AMRI and US groups respectively (Figure 1). Table 1 shows the demographics and baseline characteristics of the enrolled patients. All enrolled patients were liver cirrhotic with a median liver stiffness of 18.7 kPa; the median body mass index was 27 kg/m^2^ and diabetes was noted in 27% of patients, and while 88.8% of patients had chronic hepatitis B and/or C, none had viremia. The median follow‐up period was 33.6 months. There were no significant differences in the demographics and baseline clinical characteristics between AMRI and US groups.

**TABLE 1:** The demographics and baseline clinical characteristics of all patients, patients in the ultrasound (US) group and patients in the hepatobiliary abbreviated magnetic resonance (AMRI) group.

Variable	Total (*n* = 89)	US (*n* = 44)	AMRI (*n* = 45)	*p*
Age (year, median, IQR)	61 (54, 66)	62 (55, 65.5)	58 (53, 67)	0.802
Sex (%)	52 (58.4%)	23 (52.3%)	29 (64.4%)	0.244
Male	37 (41.6%)	21 (47.7%)	16 (35.6%)	
Female				
LS (kPa, median, IQR)	18.7 (13.1, 23.35)	19 (12.9, 24.8)	18.4 (13.2, 22.8)	0.918
BMI (kg/m^2^, median, IQR)	27.1 (23.7, 30.5)	27.1 (23.9, 30.8)	27.2 (23.5, 30.3)	0.768
Waist (cm, median, IQR)	92 (85.75, 99.5)	93 (87, 98)	92 (85, 100)	0.844
Diabetes (%)	65 (73.0%)	32 (72.7%)	33 (73.3%)	0.949
No	24 (27.0%)	12 (27.3%)	12 (26.7%)	
Yes				
Alb (g/dl, median, IQR)	4.54 (4.31, 4.72)	4.505 (4.305, 4.64)	4.59 (4.36, 4.76)	0.249
AST (IU/L, median, IQR)	33 (25, 42)	33 (25, 40)	34 (28, 42)	0.514
ALT (IU/L, median, IQR)	31 (22, 42)	28 (21.5, 43)	34 (24, 41)	0.473
BIL (mg/dl, median, IQR)	1 (0.9, 1.3)	1 (0.8, 1.25)	1.1 (0.9, 1.3)	0.242
ALP (IU/L, median, IQR)	73 (59, 88)	76 (62, 92)	66.5 (52, 86)	0.310
GGT (IU/L, median, IQR)	23 (14, 42)	23.5 (14.5, 44)	22 (14, 42)	1.000
CR (g/dl, median, IQR)	0.83 (0.69, 0.99)	0.8 (0.66, 0.975)	0.86 (0.71, 0.99)	0.305
PLT (×10^9^/L, median, IQR)	138 (104, 161)	134.5 (104, 169)	140 (104, 157)	0.809
PT‐INR (median, IQR)	1.03 (1.00, 1.06)	1.04 (1.00, 1.09)	1.03 (1.00, 1.04)	0.462
AFP (ng/dl, median, IQR)	4.1 (2.6, 5.8)	4.15 (2.8, 5.9)	4.1 (2.6, 5.8)	0.987
ALBI (median, IQR)	−3.05 (−3.22, −2.80)	−2.99 (−3.20, −2.76)	−3.09 (−3.22, −2.86)	0.498
Etiology (%)				
Non‐BC	10 (11.2%)	6 (13.6%)	4 (8.9%)	0.541
B	47 (52.8%)	20 (45.5%)	27 (60.0%)	
C	28 (31.5%)	16 (36.4%)	12 (26.7%)	
B + C	4 (4.5%)	2 (4.5%)	2 (4.4%)	
Antiviral therapy (%)				
No	1 (1.1%)	1 (2.3%)	0 (0.0%)	0.414
Yes	78 (87.6%)	37 (84.1%)	41 (91.1%)	
FU (months, median, IQR)	33.6 (23.8, 36.0)	33.7 (23.2, 36.6)	33.6 (23.8, 36.0)	0.673

Abbreviations: AFP: alpha‐fetoprotein; Alb: albumin; ALBI: albumin‐bilirubin grade; ALP: alkaline phosphatase; ALT: alanine transaminase; AST: aspartate transaminase; B: hepatitis B virus; BIL: bilirubin; BMI: body mass index; C: hepatitis C virus; CR: creatinine; FU: follow‐up; GGT: gamma‐glutamyl transferase; IQR: interquartile range; LS: liver stiffness; PLT: platelet count; PT‐INR: prothrombin time‐international normalized ratio.

### 3.2 Hepatic Nodule and HCC Occurrence

**FIGURE 2:**
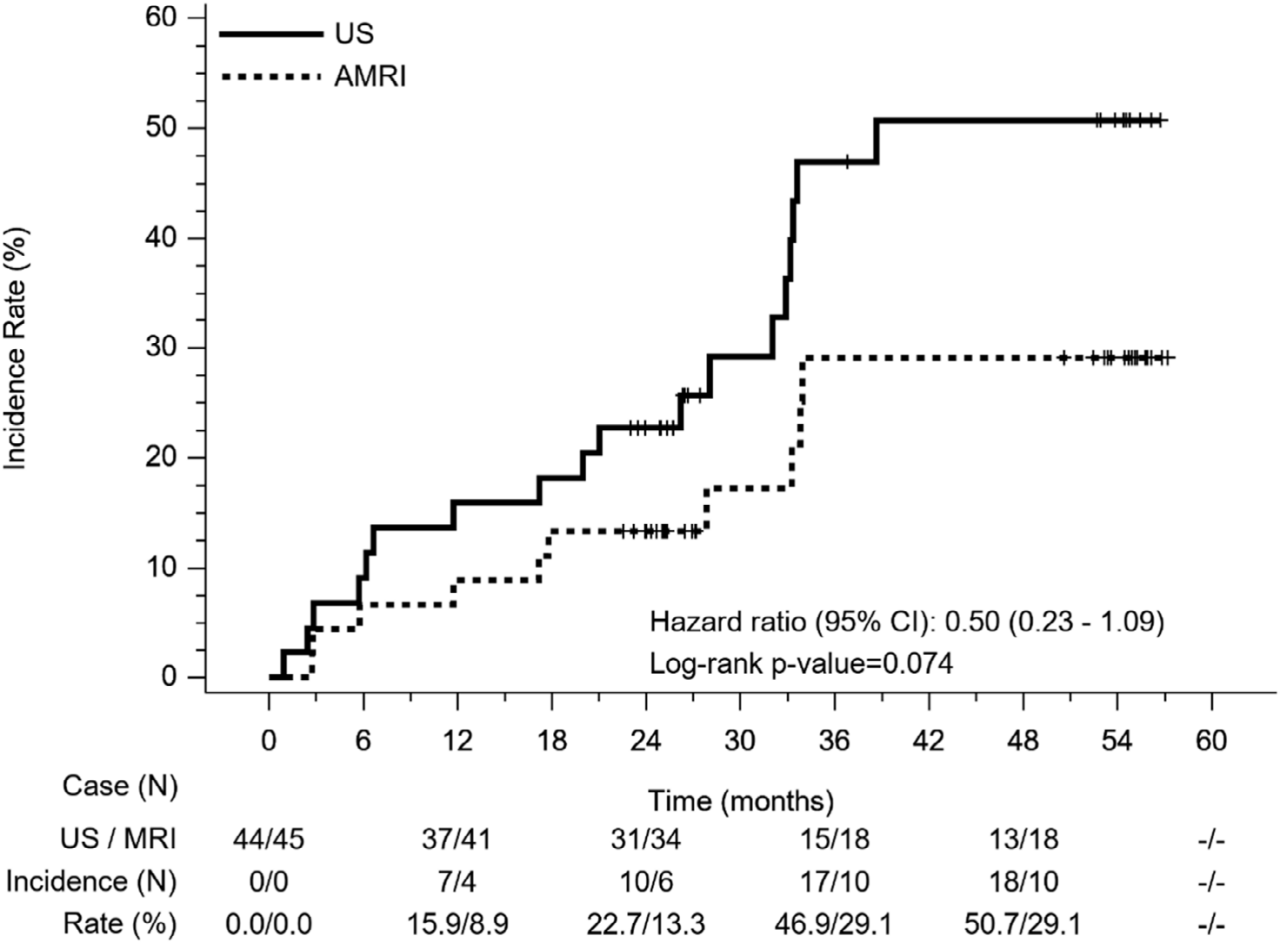
The detection of hepatic nodule in ultrasound (US) and abbreviated magnetic resonance (AMRI) surveillance groups. There is no significant difference between groups (Hazard ratio: 0.50, 95% CI: 0.23–1.09, *p* = 0.074).

In the study period, 18 and 10 patients with hepatic nodules were detected in the US and AMRI groups, respectively. There were no significant differences in the 3‐year cumulative incidence of hepatic nodules detection, which was 49.9% and 29.1% in the US and AMRI groups (Figure 2). HCC was diagnosed in six and one patients without significant difference (*p* = 0.058), while the 12‐, 24‐, and 36‐month cumulative HCC incidences were 4.5%, 5.6%, and 9.5%, respectively. While the mean HCC size was 2.4 cm in the US group, it was 1.2 cm in BCLC very early stage for the only patient in the AMRI group, while one patient had HCC in BCLC intermediate stage in the US group. Dynamic imaging, including computed tomography or MRI, was performed in 18 and 9 patients in the US and AMRI groups, respectively. Liver nodule biopsy was also performed in four and one patients in the US and AMRI groups, respectively. Significantly more diagnostic tests were performed for patients in the US group (*p* = 0.032) (Table 2).

**TABLE 2:** The hepatic nodule detection, diagnostic procedure, hepatoma (HCC) diagnosis and stage for patients in ultrasound (US) and hepatobiliary abbreviated magnetic resonance (AMRI) groups.

	AMRI group (*n* = 45)	US group (*n* = 44)	*P*
Screen tests			
US (median, IQR)	9 (9–12)	10 (8.5–12)	0.871
AMRI	2 (2–3)	—	
Liver nodule detection			
Number (%)	10 (22.2)	18 (40.9)	0.058
Diameter (cm, mean ± SD)	1.7 ± 0.8	2.6 ± 1.5	0.083
Diagnostic tests			
Dynamic image and biopsy (%)	9 (20.0)	18 (40.9)	0.032
Nodule biopsy (%)	1 (2.2)	4 (9.1)	0.203
HCC diagnosis			
Number (%)	1 (2.2)	6 (13.6)	0.058
Diameter (cm, mean + ‐SD)	1.2	2.4 ± 0.9	—
BCLC			1.000
0 (%)	1/1 (100.0)	2/6 (33.3)	
A (%)	0/1 (0.0)	3/6 (50.0)	
B (%)	0/1 (0.0)	1/6 (16.7)	
Mortality (%)	0 (0)	3 (6.8)	0.117

### 3.3 Survival

**FIGURE 3:**
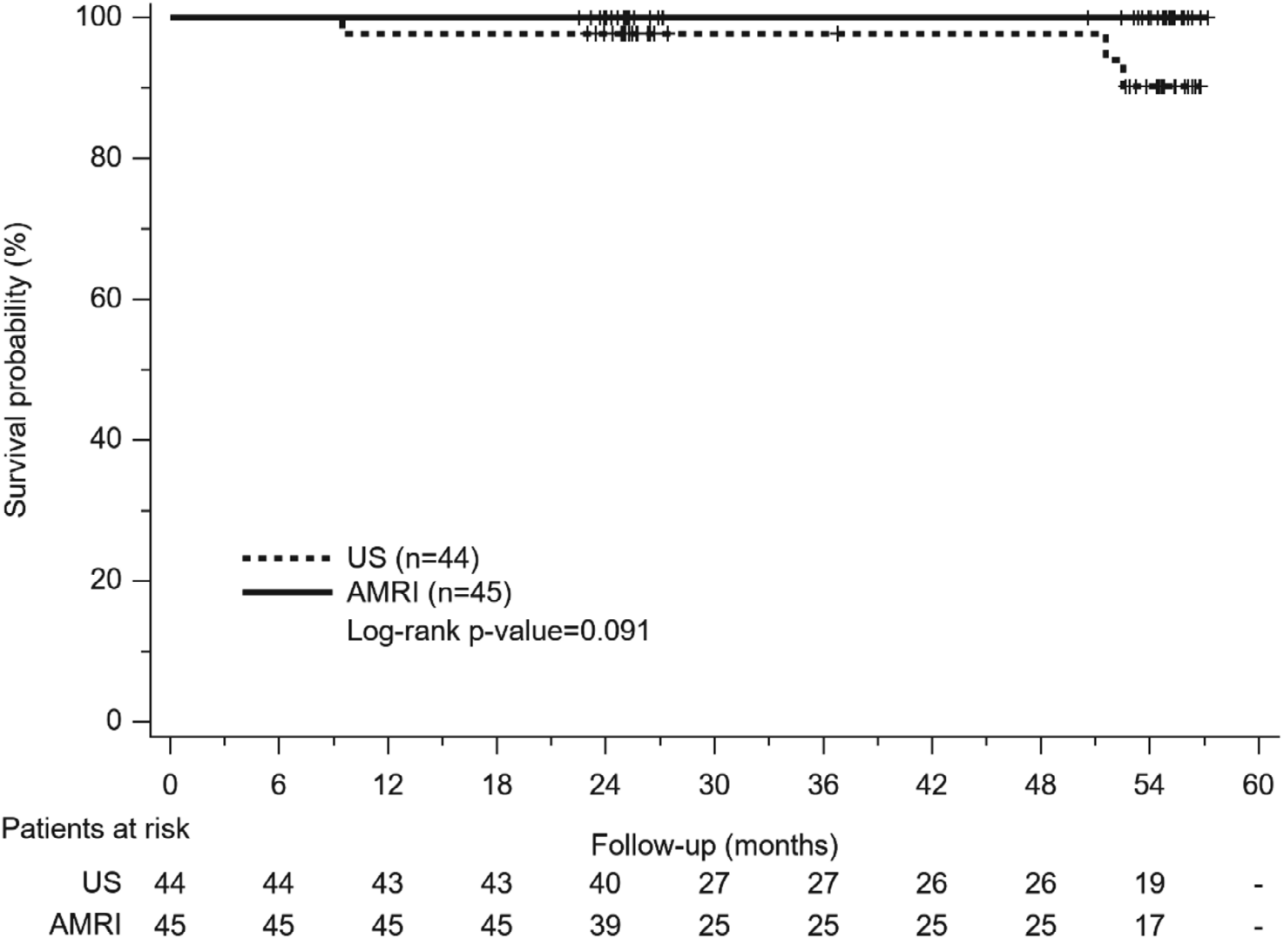
The overall survival of patients in ultrasound (US) and abbreviated magnetic resonance (AMRI) surveillance groups. There is no significant difference between groups (*p* = 0.091).

Curative treatments were performed for patients with HCC, including resection in one patient and radiofrequency ablation in six patients. While no mortality occurred in the AMRI group, three patients expired in the US group, including one of HCC progression and two of hepatic decompensations. There was no significant difference in the overall survival between the two groups (*p* = 0.091) (Figure 3).

## 4 Discussion

Recent studies have suggested that AMRI might be a feasible alternative to US and a cost‐effective strategy in HCC surveillance for high‐risk patients [11, 12, 13]. Compared with the recommended US surveillance strategy, this randomized study demonstrated that add‐on hepatobiliary AMRI performed annually to US surveillance reduced the performance of diagnostic tests and might potentially detect more patients with small or early‐stage HCC. Curative treatments were undergone for all patients with HCC developments. Our study suggested annual hepatobiliary AMRI might be added to the current US surveillance strategy in reducing the number of diagnostic tests that might cause physical and psychological harms to patients with liver cirrhosis.

Although HCC risk in cirrhotic patients varies with underlying etiology, HCC surveillance with semiannual US with AFP has been recommended for cirrhosis patients with annual HCC incidence ⩾ 1.0% in one professional society [4]. While antiviral therapy might reduce HCC risk, the risk of HCC remains high in patients with viral hepatitis‐related cirrhosis [15, 16]. In this study, most of the patients enrolled were hepatitis B or C virus‐related cirrhosis in complete or sustained viral response. The enrolled patients were at intermediate or high risk of HCC with a 3‐year cumulative incidence of 9.5% in which the surveillance strategy of AMRI alternating with US or biannual AMRI was recommended [14]. Additionally, our study demonstrated that the cirrhosis criteria generated by combining liver stiffness and US findings were useful in the identification of high‐risk patients who would benefit from HCC surveillance following antiviral therapy.

Alternative surveillance tools for HCC surveillance have been suggested for those prone to inadequate US qualities and worse surveillance test performance [7, 17]. In a prospective survey in patients with cirrhosis, AMRI was the most preferred surveillance tool for its high sensitivity in early HCC detection and convenience [18]. The factors associated with poor US quality include obesity, severe cirrhosis, and alcohol‐ or metabolic dysfunction‐related cirrhosis [7]. In this study, most patients were obese and/or had high waist circumference, where alternative modalities might be necessary to increase surveillance performance. While AMRI was the preferred tool, the protocol and strategy in the surveillance were not evaluated prospectively in HCC surveillance.

There were three AMRI protocols including non‐contrast, dynamic contrast‐enhanced, and hepatobiliary with various sequences, advantages, and disadvantages [14]. A retrospective study has demonstrated that hepatobiliary AMRI with the sequences of diffusion‐weighted, T2‐weighted, and hepatobiliary‐phase imaging had only a rather inadequate 10% imaging quality and higher sensitivity than US [19]. In a recent prospective cohort study, annual non‐contrast AMRI had a higher diagnostic yield than biannual US; however, without significantly higher sensitivity for HCC [20]. While prospective trials are ongoing [21], this randomized study has shown that add‐on hepatobiliary AMRI annually to the recommended US surveillance might potentially detect small and early‐stage HCC and be a promising strategy to overcome inadequate quality and low sensitivity of US. Large studies will be necessary to confirm the benefit of our strategy in HCC surveillance.

Although HCC surveillance was beneficial in detecting early‐stage HCC for curative treatment, potential physiological, financial, and psychological harms might arise from the screening and diagnostic tests. About 20% of patients with indeterminate nodules requiring computed tomography/MRI and 27.5% of cirrhotic patients undergoing HCC surveillance both experienced physical harms from dynamic imaging and biopsy [22]. Compared with the AMRI group, significantly more diagnostic tests including dynamic imaging and biopsy were performed in the US surveillance group in our study; nevertheless, no statistical differences in HCC confirmations, false positive, and negative rates in the screening and diagnostic tests between the two groups were found.

Although the recall policy of abnormal US imaging from practice guidelines is generally accepted [4], the study protocol was proposed based on clinical practice in which the physicians performed diagnostic tests at their judgment without conforming the recall policy completely; consequently, the results might be explained by the physician's confidence in identifying the false‐positive abnormal US images with the help of add‐on hepatobiliary AMRI, thereby reducing the performance of dynamic imaging and biopsy. A large study with the enrollment of more patients might be necessary to validate this finding.

While it remains a controversial issue, HCC surveillance with the recommended semiannual US improved patient survival in meta‐analysis studies [4], there are limited studies on the impact on HCC survival for different surveillance strategies using MRI. In a retrospective cohort study, intermittent GA‐enhanced MRI replacement of US surveillance improved early‐stage HCC detection and overall survival [23]. Whether the surveillance strategy with AMRI improves HCC survival is unknown. In this study, there were more mortalities, with one being due to HCC progression in the US groups; however, without statistical significance. In diagnostic settings, GA‐enhanced MRI had been demonstrated to change HCC stage and reduce mortality owing to the high sensitivity of the HBP phase in HCC detection [24, 25]; accordingly, HCC surveillance with hepatobiliary AMRI might have the potential and cost‐effectiveness in reducing mortality. Long‐term follow‐up with a larger cohort is necessary to further validate these findings.

In summary, our study demonstrated that HCC surveillance with add‐on hepatobiliary AMRI annually on recommended US might reduce the frequency of diagnostic tests that caused physical and psychological harm to patients with compensated cirrhosis.

## Ethics Statement

This study was approved by the Institutional Review Board of Kaohsiung Chang Gung Memorial Hospital and carried out in compliance with the Helsinki declaration.

## Conflicts of Interest

The authors declare no conflicts of interest.

## Data Availability

The data that support the findings of this study are available on request from the corresponding author. The data are not publicly available due to privacy or ethical restrictions.
